# The Insufficiency of Sanatoria for the Consumptive

**Published:** 1903-06-27

**Authors:** 


### The Insufficiency of Sanatoria for the Consumptive.

Hygienic management affords the most effective known means of combating pulmonary consumption. The recognition both by the medical profession and all thinking persons of the great advantages secured by the adoption of so-called "open-air" treatment, and the speedy perception of the fact that the necessary requirements can be best obtained and most effectually maintained in a specially constructed and specifically directed institution, has led to the rapid upspringing of sanatoria in all civilised countries. In Great Britain and Ireland many very excellent institutions now exist where consumptives may obtain efficient means for placing themselves under the best possible conditions for procuring arrest of the morbid processes and building up high powers of tissue resistance. But for the most needy and necessitous cases there is in this country an altogether deplorable insufficiency of suitable sanatoria. It is very necessary at a time when much of the old pessimism is passing, and at least in some quarters a perhaps too optimistic view prevails, that the seriousness of the present position should be faced without delay. In some respects the case of the poor consumptive was never more pitiable. He has held out before him in all the allurements of hope and desire a means of escape from a death-directing disease only to find on attempting to secure this much-lauded and desirable relief that he has to contend with almost endless discouragement and oftentimes fatal delay before succour arrives. In many ways the difficulty of providing for the necessitous poor afflicted with consumption seems to increase rather than diminish.

General hospitals, convalescent homes, and many private and philanthropic institutions for the care of the sick poor which formerly were accustomed to provide for considerable numbers of consumptives now, no doubt very properly, close their doors on such cases and send them away to search for means whereby their condition may be made known to those responsible for the administration of the very few institutions providing open-air treatment for the indigent. The present objectionable and altogether unscientific method of requiring introduction by means of a subscriber's "letter" often leads to serious delay and much weariness, disappointment, and needless trouble. But even when the claims of an applicant have been received and his or her suitability for treatment made clear by thorough medical examination, most of our free institutions are so overwhelmed with patients seeking relief that several months, even up to half a year, may elapse before the sufferer can be received, and when the turn for admission to the sanatorium comes the patient is almost invariably much worse, in many instances the disease has advanced to such an extent as to make arrest practically impossible, and in not a few cases the state is such as after all this waiting and hope deferred to make rejection necessary. This condition of affairs raises questions of profound ethical importance. The present situation is indeed deplorable and pitiable in the extreme. It is cruel irony for medical men to have to go on urging the necessity of an early recognition of the disease and the application of hygienic measures at the earliest possible moment, when for most of the large numbers of the poor effective treatment in the initial stage is almost an impossibility.

In the well-founded hope aroused by the striking results of sanatorium treatment there is danger of losing sight of the seriousness of the disease. Pulmonary tuberculosis is not a morbid condition which may be viewed lightly, and those who talk glibly of a "cure" in a few months but little understand the situation. It is very necessary that such light and flippant views of pathology should be discouraged and discarded; they are fruitful of much evil. Philanthropic effort alone cannot adequately cope with the urgent demands of the consumptive poor. Our Poor Law institutions are at present in most instances quite unfitted for the effective management of consumptives according to the best hygienic methods. It is becoming abundantly clear that collective public action must be taken, and that speedily, for it is only by adequate and efficient provision for the dangerous indigent consumptive that the healthy can be protected from swelling the ranks of the tuberculous sufferers.

